# A perfusion study of the handling of urea and urea analogues by the gills of the dogfish shark (*Squalus acanthias*)

**DOI:** 10.7717/peerj.33

**Published:** 2013-02-12

**Authors:** Chris M. Wood, Hon Jung Liew, Gudrun De Boeck, Patrick J. Walsh

**Affiliations:** 1 Department of Biology, McMaster University, Hamilton, Ontario, Canada; 2 Bamfield Marine Sciences Centre, Bamfield, BC, Canada; 3 Division of Marine Biology and Fisheries, Rosenstiel School, University of Miami, Miami, Florida, USA; 4 Institute of Tropical Aquaculture, Universiti Malaysia Terengganu, Kuala Terengganu, Malaysia; 5 Department of Biology (SPHERE), University of Antwerp, Belgium; 6 Department of Biology, University of Ottawa, Ottawa, Ontario, Canada

**Keywords:** Elasmobranchs, Thiourea, Acetamide, Perfused head, Urea retention

## Abstract

The branchial mechanism of urea retention in elasmobranchs was investigated using an *in vitro* isolated-perfused head preparation, as well as *in vivo* samples, in the spiny dogfish shark. Both *in vivo* and in control saline perfusions containing 350 mmol L^−1^ urea, calculated intracellular urea concentrations in gill epithelial cells were close to extracellular concentrations. Urea efflux to the external water fell only non-significantly, and calculated gill intracellular urea concentration did not change when perfusate urea concentration was reduced from 350 to 175 mmol L^−1^ with osmotic compensation by 175 mmol L^−1^ mannitol. However, when the urea analogues thiourea or acetamide were present in the perfusate at concentrations equimolar (175 mmol L^−1^) to those of urea (175 mmol L^−1^), urea efflux rates were increased 4-fold and 6.5-fold respectively, and calculated gill intracellular urea concentrations were depressed by about 55%. Analogue efflux rates were similar to urea efflux rates. Previous studies have argued that either the basolateral or apical membranes provided the limiting permeability barrier, and/or that a back-transporter on the basolateral membranes of gill cells is responsible for urea retention. The present results provide new evidence that the apical membrane is the limiting factor in maintaining gill urea impermeability, and raise the prospect that a urea back-transporter, which can be competitively inhibited by thiourea and acetamide, operates at the apical membrane.

## Introduction

The ability of elasmobranch fishes to retain very high concentrations of the nitrogenous waste urea in their body fluids for the purpose of osmoregulation has fascinated biologists for more than a century (Staedler & Frerichs, 1858; Smith, 1929; Smith, 1936). Of particular interest is the ability of the gills to maintain extremely low effective permeability to urea while allowing for O_2_ and CO_2_ exchange at comparable rates to teleost fishes. For example, Pärt, Wright & Wood (1998) have made the instructive calculation that given the high blood plasma concentrations of urea (typically 300–400 mmol L^−1^) in elasmobranchs, if their gill urea permeability was comparable to that of teleosts, urea efflux would be about 10,000 µmol kg^−1^ h^−1^ , about 40 fold higher than typical values measured *in vivo* (Wood, Pärt & Wright, 1995).

There have been only a few studies on the mechanism of urea retention at the gills, all of them on the spiny dogfish *Squalus acanthias*. Boylan (1967) concluded that the lipid membranes in the gills were of unusual composition, resulting in exceptionally low permeability to urea, a conclusion based largely on changes in urea efflux rate with temperature. However he did raise (and dismiss) the possibility of a retention mechanism based on active transport.

Nevertheless, the latter idea gained some credence from the experiments of Wood, Pärt & Wright (1995), who infused the urea analogues thiourea and acetamide into intact dogfish so as to achieve blood plasma analogue concentrations of no more than 15% those of urea, yet observed 20–40% increases in branchial urea efflux rates. Since these analogues are simple competitive inhibitors in many urea transport systems of higher vertebrates (Marsh & Knepper, 1992), Wood, Pärt & Wright (1995) suggested that they acted to inhibit a “back-transport” retention mechanism in the gills, resulting in greater urea efflux.

Further support for both ideas was provided by the perfused dogfish head studies of Pärt, Wright & Wood (1998). Based on [^14^C]urea “washout” experiments, these workers identified the apical membranes of gill cells as 14-fold less permeable than the basolateral membranes, and also reported that phloretin, a general non-competitive blocker of urea transport in many systems, greatly exacerbated urea efflux to the water. This was interpreted as a result of inhibition of an active urea back-transporter on the basolateral membrane, but curiously, thiourea, at equimolar concentration to urea, had no effect.

The presence of an active urea back-transport mechanism and its inhibition by phloretin, were confirmed in gill basolateral membrane vesicle experiments by Fines, Ballantyne & Wright (2001). Again, thiourea, as well as acetamide, had no significant inhibitory effects, even though presented at 148-fold greater concentration than urea. However these workers also demonstrated that the gill basolateral membranes had an exceptionally high cholesterol-to-phospholipid molar ratio, which they thought would result in exceptionally low urea permeability. In model calculations, they suggested that only 6% of the difference in gill urea permeability between elasmobranchs and teleosts was due to the back-transporter, and that 94% was due to the impermeability of the basolateral membranes, not the apical membranes as argued by Pärt, Wright & Wood (1998).

The situation was further complicated by direct measurements of urea permeabilities in apical and basolateral gill membrane vesicles by Hill et al. (2004). These workers found that the values for both were not exceptionally low but rather about the same in elasmobranchs and teleosts, and at least 10-fold higher than needed to explain urea impermeability *in vivo*. Since the basolateral permeability was slightly higher than the apical permeability, and the apical membrane surface area would be much less, they agreed with Pärt, Wright & Wood (1998) that the apical membrane would be the more effective barrier, but concluded that some other mechanism was needed to explain urea impermeability *in vivo*.

While there were many differences, a common finding in all of the *in vivo* studies (Boylan, 1967; Wood, Pärt & Wright, 1995; Pärt, Wright & Wood, 1998) was that there appeared to be little or no relationship between extracellular urea concentration and urea efflux rate across the gills. Pärt, Wright & Wood (1998) hypothesized that this could be explained if the back-transporter on the basolateral membrane kept intracellular urea levels much lower than those in plasma. This idea was supported by the finding of Fines, Ballantyne & Wright (2001) that the *K*_*m*_ of the basolateral membrane urea transporter was only 10 mmol L^−1^, suggesting that its normal operating concentration was far below the 300–400 mmol L^−1^ normally present in blood plasma (Robertson, 1975; Wood, Pärt & Wright, 1995; Kajimura et al., 2006). If true, then the urea gradient across the apical membrane would be >10-fold lower than previously supposed, and the apical permeability might be low enough to explain *in vivo* efflux rates (Pärt, Wright & Wood, 1998; Fines, Ballantyne & Wright, 2001; Hill et al., 2004).

With this rather confounded background in mind, we revisited the perfused dogfish head preparation of Pärt, Wright & Wood (1998) to address the following issues using radiolabel techniques. We hypothesized that the apparent lack of effect of thiourea (and acetamide) was due to methodological issues in the original work, and would be demonstrated with a longer perfusion period. We also measured gill tissue concentrations of urea *in vivo*, and concentrations of urea, thiourea, and acetamide in the perfused gill tissue, as well as extracellular and intracellular space in the latter. We hypothesized that the analogues would increase urea efflux rates, would displace urea from the gill cells, and would be excreted at comparable rates to urea when presented at equimolar concentrations in the perfusate. We also hypothesized that gill cell urea concentration would be buffered from changes in extracellular urea concentration, and would be much lower than the latter. Most of these hypotheses were confirmed, but one disproven, leading to a new proposal for how urea is retained in elasmobranch gills.

## Materials and methods

### Experimental animals

Experiments were performed at Bamfield Marine Sciences Centre (BMSC) over several summers (July–August) on 56 male spiny dogfish sharks (*Squalus acanthias suckleyi*; 0.3–1.7 kg) collected locally from Barkely Sound by trawling. Only males were used for purposes of population conservation, as females were invariably pregnant. [Note that Ebert et al. (2010) have recently proposed that these north-east Pacific elasmobranchs are a separate species (*Squalus suckleyi*) distinct from *Squalus acanthias*]. The fish were held in a large 151,000-L tank at seasonal photoperiod, served with running seawater at the experimental temperature (12 ± 1 °C), salinity (30 ± 2 ppt) and pH (7.95 ± 0.10) for 2–8 weeks prior to experimentation. During this period they were fed every 3–4 days with a 3% ration of dead hake (*Merluccius productus*), but removed to smaller 1000-L tanks for 1–2 weeks fasting prior to experimentation, under the same conditions. Gill tissue and plasma samples were taken from 9 of the animals sacrificed for other studies, in order to provide *in vivo* reference values; the remainder were used for *in vitro* perfused head preparations. The *in vivo* samples were taken from deeply anaesthetized animals, as described below for the perfused head preparations. All procedures followed Canada Council for Animal Care guidelines and were approved by BMSC and McMaster University Animal Care Committees (Animal Ethics Research Board of McMaster University AUP # 09004-10).

### Perfused head preparation

Methods followed those developed by Pärt, Wright & Wood (1998), with minor modifications. Before surgery, the fish were injected with 5000 i.u. sodium heparin (Sigma-Aldrich, St. Louis, MO, USA) via the caudal vein and left for 15–20 min. The fish was then deeply anaesthetized in MS-222 (0.2 g L^−1^; Syndel Laboratories, Qualicum Beach, BC, Canada) and decapitated posterior to the pectoral fins. The brain was pithed via the spinal cord. The ventral aorta was cannulated with Clay-Adams PE-160 tubing (Becton Dickinson, Franklin Lakes, NJ, USA), and the dorsal aorta with PE60 or PE160 depending on fish size; both ventral and dorsal aortic catheters were filled with the appropriate saline (see below). A thin latex rubber sheet (dental dam) placed anterior to the pectoral fins was then used to seal the head into a 1.8 L-external reservoir made from a plastic bottle, and internal perfusion and external irrigation were commenced immediately (time = 0 min).

The external reservoir was filled with approximately 1.0 L of sea water which was continuously gassed with O_2_ and recirculated via an external submersible pump (Little Giant, Oklahoma City, OK, USA) connected to the bottom of the reservoir. The pump, controlled by a rheostat, was set to irrigate the gills at a rate of 2.5 L min^−1^ via soft silastic tubing (0.5 cm i.d.) inserted via a Y-tube into both spiracles. A Model 1100 cardiac pump (Harvard Apparatus, Holliston, MA, USA) set to a working mode of 1/3 systole, 2/3 diastole and a heart rate of 40 beats min^−1^ was employed for perfusion via the ventral aortic catheter with the appropriate saline which had been pre-gassed as described below. The stroke volume was adjusted for each preparation so as to create a perfusion rate of 20 ml min^−1^ kg whole body^−1^. The output of the cardiac pump passed first through an inverted 25-ml Erlenmeyer flask containing a 5-ml air–space which served as a “*windkessel*” to reduce pulsatility and to trap inadvertent bubbles, and then past a T-junction connected to a P23BB pressure transducer (Statham Instruments, Oxnard, CA, USA) linked to a Harvard amplifier-oscillograph system so as to continuously monitor the input perfusion pressure to the ventral aorta. The T-junction also served as a sampling point for ventral aortic perfusate. The external reservoir pump and the perfusion reservoir were both immersed in flowing seawater baths so as to maintain 12 °C. Only one saline treatment was used for each preparation, for a 45 min perfusion period.

Samples of external water (10 ml), inflowing ventral aortic perfusate (0.1 ml) from the T-junction, and outflowing perfusate (0.1 ml) directly from the dorsal aortic catheter, were taken at 5 min intervals from time 0 to 45 min. These samples were then aliquotted immediately for radioactivity measurements and frozen for later measurements of urea. At the end of 45 min, perfusion and irrigation were stopped, the head was removed, and the exact water volume of the external reservoir was measured. The pressure drop across the catheter at the same cardiac pump output was measured at the T-junction so as to calculate the true ventral aortic input pressure. The gills were removed by dissection, blotted dry, and the soft tissue of the gill epithelium was sampled by gently scraping the surface with a glass slide. Three samples of approximately 0.5 g were immediately added to tared tubes containing 1 ml of ice-cold 8% HClO_3_ . The tubes were reweighed to determine the exact weight of gill tissue, then allowed to sit on ice for 3 h with intermittent vortexing. Thereafter the tubes were centrifuged at 13,000G for 2 min and the supernatant aliquotted for radioactivity measurements and frozen for later measurements of urea. Additionally two gill tissue samples of approximately 0.3 g were weighed out into aluminum dishes for determination of water content.

### Perfusion salines

The basic saline (Control saline 1) contained 350 mmol L^−1^ urea and was identical to that used by Pärt, Wright & Wood (1998) – see Table 1 in that paper – except that the trimethyl amine oxide (TMAO) concentration was set to 85 mmol L^−1^ rather than 15 mmol L^−1^ to better represent *in vivo* values (Schmidt-Nielsen, Truniger & Rabinowitz, 1972; Robertson, 1975; Kajimura et al., 2006). All other salines were modifications of this. In Control saline 2, urea concentration was reduced to 175 mmol L^−1^, with osmotic balance achieved by addition of 175 mmol L^−1^ mannitol. For treatments with the urea analogues thiourea or acetamide, the saline contained 175 mmol L^−1^ urea and 175 mmol L^−1^ of the respective analogue. All salines were filtered through a 0.22 µm Millipore filter prior to use, and were equilibrated with a precision gas mixture of 0.25% CO_2_ in air to yield a PCO_2_ of about 1.9 mm Hg. The pH was set to 7.7–7.8. Diffusion of the gas through silastic tubing rather than direct bubbling was employed to avoid foaming of the bovine serum albumen component.

**Table 1:** The water content of gill tissue and its distribution.

Treatment	Water (kg H_2_O.kg tissue^−1^ )
Thiourea (175 mmol L^−1^) + Urea (175 mmol L^−1^)	0.8522 ± 0.0037^c^ (6)
Acetamide (175 mmol L^−1^) + Urea (175 mmol L^−1^)	0.8617 ± 0.0036^c^ (6)
*In vivo*	0.8273 ± 0.0043^b^ (9)
Control Saline 1 (350 mmol L^−1^ Urea)	0.8199 ± 0.0067^ab^ (10)
Control Saline 2 (175 mmol L^−1^Urea + 175 mmol L^−1^ Mannitol)	0.8075 ± 0.0025^a^ (5)
Extracellular Fluid Volume (ECFV)	0.5230 ± 0.0023 (5)
Intracellular Fluid Volume (ICFV)	0.2845 ± 0.0023 (5)

**Notes.**

Means ± 1SEM (N). Means not sharing the same letters are significantly different (*P* < 0.05). ECFV and ICFV were measured only for the Control Saline 2 treatment.

In all of the experiments, urea efflux was calculated from the appearance of “cold” urea in the external seawater, as measured by chemical assay. The same assay was used to measure urea concentration in the gill tissue. However there is no assay of comparable sensitivity for the urea analogues. Therefore in order to evaluate whether radiotracer methodology would be suitable, in 6 preparations perfused with Control saline 1, [^14^C]urea (Amersham, Little Chalfont, UK; 59 mCi mmol^−1^ ) was added to the perfusion medium at a concentration of 50 µCi L^−1^ to allow simultaneous measurements by both “hot” and “cold” techniques. Based on these results, [^14^C]thiourea (50 µCi L^−1^ ; Sigma-Aldrich; 30–60 mCi mmol^−1^) and [^14^C]acetamide (50 µCi L^−1^ ; NEN-Dupont, 58 mCi mmol^−1^) were added to the perfusion saline in the respective analogue trials. In order to measure extracellular and intracellular spaces, 10 µCi L^−1^ of the extracellular space marker [^3^H]methoxy inulin (Perkin-Elmer, Woodbridge, ON, Canada; 2500 mCi mmol^−1^) was added to the saline in 5 preparations perfused with Control saline 2. This added a negligible amount of “cold” methoxy inulin ( < 10^−5^ mmol L^−1^) to the perfusate.

### Analytical techniques

Urea in sea water, perfusate, and gill extract samples was measured by the colorimetric diacetyl monoxime assay of Rahmatullah & Boyde (1980). All radioactivity measurements were made on a LS6500 scintillation counter (Beckman Coulter, Fullerton, CA, USA). Water samples (5 ml) for determination of [^14^C]urea, [^14^C]thiourea, or [^14^C]acetamide radioactivity were added to 10 ml of Aqueous Counting Scintillant (ACS) (Amersham, Little Chalfont, UK). Perfusate samples (50 µl) and gill extract samples (100 µl) were diluted to 5 ml with sea water and similarly added to 10 ml ACS. Quench correction was performed by an onboard program using the external standard (Beckman H-# system) and checked by internal standardization. For determination of [^3^H]methoxy inulin radioactivity, 0.8 ml of gill extract and 1.0 ml of perfusate were diluted to 2 ml with seawater and added to 4 ml of ACS. The quench in each sample was corrected by internal standardization. Water content of gill samples was determined gravimetrically by drying at 60 °C for three days.

### Calculations and statistics

Mean urea efflux rates were calculated from the slopes of the appearance curves of urea in the external sea water, factored by time, total fish body weight, and the measured volume of the external reservoir. In the case of radiolabeled compounds ([^14^C]urea, [^14^C]thiourea, and [^14^C]acetamide), the slope of the appearance curve of radioactivity (dpm h^−1^) was additionally factored by the mean specific activity (i.e. dpm µmol^−1^ ) of venous (i.e. input) perfusate samples over the same period. Measurements of gill tissue concentrations of urea, thiourea, and acetamide by [^14^C] radioactivity were based on the radioactivity of gill extracts (after correction for dilution), factored by the mean specific activity (i.e. dpm µmol^−1^ ) of venous and arterial perfusate samples taken at the end of the perfusion period. Concentrations were expressed both per kg tissue and per kg tissue water, as noted in figures.

The extracellular fluid volume of gill tissue was calculated as:

\begin{eqnarray*} \text{ECFV}=\frac{\text{Tissue }[{\text{}}^{3}\mathrm{H}]\text{methoxy inulin (dpm. kg }{\mathrm{tissue}}^{-1})}{\text{Perfusate }[{\text{}}^{3}\mathrm{H}]\text{methoxy inulin (dpm. kg perfusate }{\mathrm{H}}_{2}{\mathrm{O}}^{-1})} \end{eqnarray*}

where the numerator was based on radioactivity measurements in gill extracts, corrected for dilution, and denominator was the mean of arterial and venous radioactivity measurements at the end of the perfusion period. Intracellular fluid volume (ICFV) was calculated as the difference between total tissue water (kg H_2_O. kg tissue^−1^ ) and ECFV (kg H_2_O. kg tissue^−1^ ).

Data have been expressed as means ± 1 SEM, where *N* = number of animals. Data were checked for normality and homogeneity of variance, and in a few cases log or arc-sine transformed to achieve this. Means were compared by Student’s paired or unpaired Student’s t-test (two-tailed) as appropriate, or in the case of multiple comparisons, by 1-way ANOVA followed by the Fisher’s LSD test. Regression lines were fitted by least squares, and the significance of Pearson’s correlation coefficient (*r*) assessed. A significance level of *P* < 0.05 was used throughout.

## Results

Ventral aortic input pressure was unaffected by the experimental treatments, but increased significantly by about 15% over the course of the 45 min perfusions from 23.7 ± 1.2 to 27.2 ± 1.3 mm Hg (*N* = 44). Efflux rates of urea and urea analogues into the external recirculating water were generally low and variable in the first 10 min, but stabilized thereafter, so rates were calculated from regression lines fitted to the 10–45 min data points.

### “Cold” urea versus “hot” [^14^C]urea efflux rates and gill tissue concentrations

**Figure 1:**
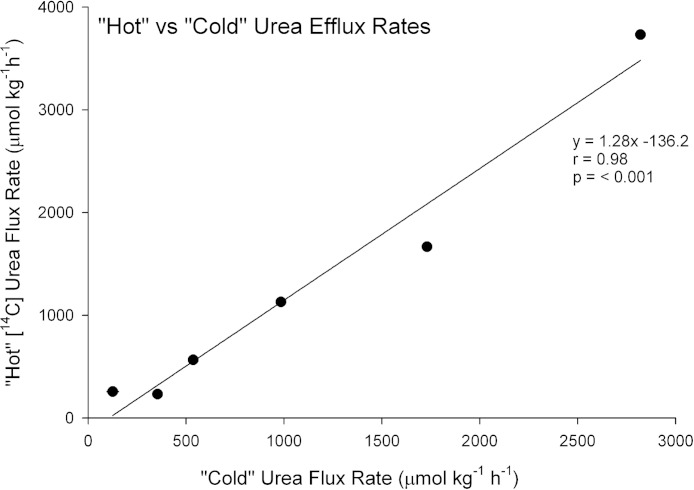
Validation of the radiotracer method. The relationship between urea efflux rate measured by [^14^C]urea radiotracer appearance (“hot”) versus urea efflux rate measured by chemical assay of urea appearance (“cold”) in the perfused head preparation of the dogfish shark (*N* = 6).

There was a strong linear relationship (*Y* = 1.28*X*−136.2, *N* = 6, *r* = 0.98, *P* < 0.001) between the efflux rate of “hot” [^14^C]labeled urea (*Y*) and the efflux rate of “cold” urea (*X*), measured simultaneously on the same preparations (Fig. 1). The relationship covered a wide range of flux rates (100–3000 µmol kg^−1^ h^−1^ ) representative of the range of urea and analogue fluxes recorded in subsequent experiments, and the slope was not significantly different from 1.0. Additionally, gill tissue urea concentration measured chemically (i.e. “cold”) in these 6 preparations was 309.5 ± 18.6 mmol kg^−1^ and by [^14^C]urea radioactivity (i.e. “hot”) was 299.0 ± 17.9 mmol kg^−1^, not significantly different. These data therefore validate the use of the radiolabel methodology for subsequent measurements of the fluxes and gill tissue concentrations of the analogues, which could not be quantified by “cold” techniques.

### Urea and analogue efflux rates

**Figure 2:**
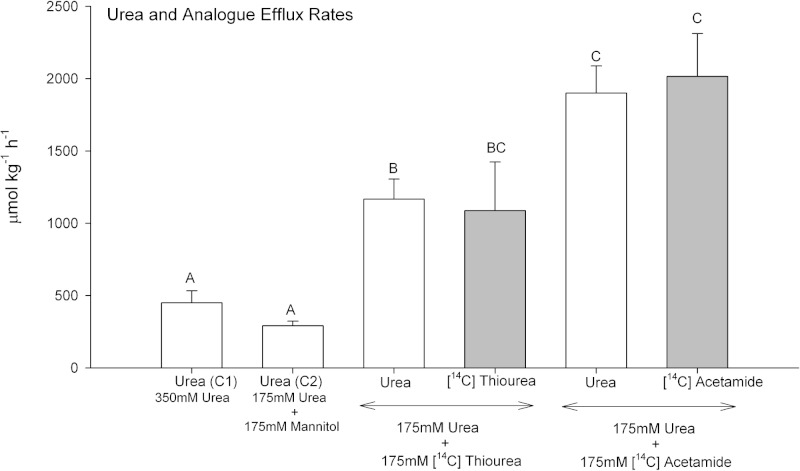
Urea and analogue efflux rates in the perfused head preparation of the dogfish shark. Open bars represent urea efflux rates; shaded bars represent analogue efflux rates. The treatment (perfusion saline) is noted below the bars: Control saline 1–350 mmol L^−1^ urea (*N* = 12); Control saline 2–175 mmol L^−1^ urea + 175 mmol L^−1^ mannitol (*N* = 5); 175 mmol L^−1^ urea + 175 mmol L^−1^ [^14^C]thiourea (*N* = 6); 175 mmol L^−1^ urea + 175 mmol L^−1^ [^14^C]acetamide (*N* = 6). Means ± 1 SEM. Means bearing the same letters are not significantly different (*P* < 0.05).

When dogfish gills were perfused with Control saline 1 containing 350 mmol L^−1^ urea, urea efflux rates averaged about 450 µmol kg^−1^ h^−1^ (Fig. 2). In separate preparations where the saline [urea] was reduced by 50% to 175 mmol L^−1^ (Control 2; osmotically compensated with 175 mM mannitol), the urea efflux rate fell by only 35%, a difference which was not significant. However, when 175 mmol L^−1^ thiourea was presented together with 175 mmol L^−1^ urea, the urea efflux rate exhibited a 4-fold increase relative to 175 mmol L^−1^ urea alone, and a 6.5-fold increase when 175 mmol L^−1^ acetamide was presented together with 175 mmol L^−1^ urea. Both of these increases were highly significant. In each case, the simultaneously measured [^14^C] analogue efflux rate was approximately equal to the urea efflux rate.

### Gill tissue urea and analogue concentrations

**Figure 3:**
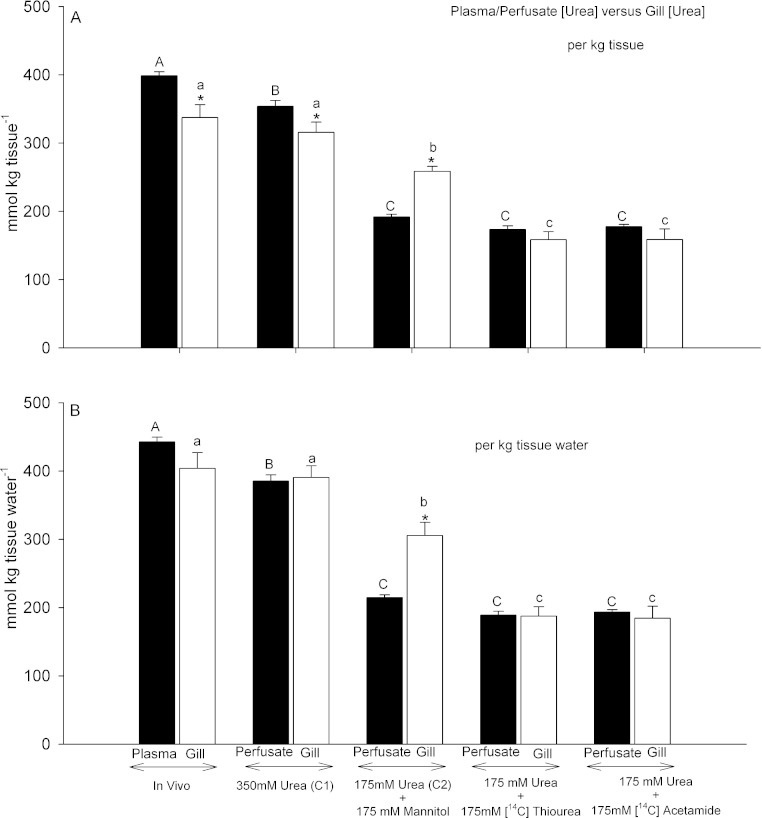
Urea concentrations in plasma or perfusate versus those measured simultaneously in gill tissue. Black bars represent concentrations in the plasma or perfusate; open bars represent concentrations in the gill tissue. In (A), concentrations are expressed on a per kg tissue or plasma/perfusate basis; in (B) concentrations are expressed on a per kg tissue H_2_O or plasma/perfusate H_2_O basis. The treatment is noted below the bars: In vivo (*N* = 9); Control saline 1–350 mmol L^−1^ urea (*N* = 10); Control saline 2–175 mmol L^−1^ urea + 175 mmol L^−1^ mannitol (*N* = 5); 175 mmol L^−1^ urea + 175 mmol L^−1^ [^14^C]thiourea (*N* = 6); 175 mmol L^−1^ urea + 175 mmol L^−1^ [^14^C]acetamide (*N* = 6). Means ± 1 SEM. Plasma/perfusate means bearing the same capital letters are not significantly different (*P* < 0.05); gill tissue means bearing the same lower case letters are not significantly different (*P* < 0.05); asterisks indicate gill tissue mean significantly different (*P* < 0.05) from corresponding plasma/perfusate mean.

When expressed on a per kg tissue or kg plasma basis, gill tissue [urea] at the end of the 45 min perfusion with Control saline 1 (350 mmol L^−1^) was about 90% of perfusate [urea], a significant difference (Fig. 3A). By way of comparison, in gill tissue sampled from intact dogfish, gill [urea] was about 85% of plasma urea, again a significant difference. Both *in vivo* values were slightly higher than in the perfused preparations, significant only for the plasma *versus* perfusate concentrations. However when the urea concentrations were expressed on a per kg tissue H_2_O and per kg perfusate or plasma H_2_O basis, there were no longer any plasma or perfusate *versus* gill tissue differences, indicating equilibration between the compartments for Control saline 1 and *in vivo* treatments (Fig. 3B).

This was not the case for gills perfused with Control saline 2 (175 mmol L^−1^ urea, osmotically compensated with mannitol). On a per kg tissue or perfusate basis, gill [urea] was 34% higher than perfusate [urea] (Fig. 3A), and this difference increased to 42% when the data were expressed on a per kg tissue H_2_O and per kg perfusate H_2_O basis (Fig. 3B). Thus gill [urea] tended to be maintained, falling by only about 22% despite a 45 min perfusion with Control saline 2; it did not decline in proportion to the 50% difference in perfusate [urea] between Control saline 1 and Control saline 2.

A very different pattern was seen when 175 mmol L^−1^ thiourea or 175 mmol L^−1^ acetamide was presented together with 175 mmol L^−1^ urea. Gill [urea] declined by approximately 50% relative to the control saline 1 treatment, and was about equal to perfusate [urea] regardless of whether the data were expressed on a per kg tissue basis (Fig. 3A) or a per kg tissue H_2_O basis (Fig. 3B). These tissue urea concentrations were substantially lower than in the Control saline 2 treatment, despite identical perfusate urea concentrations (Figs. 3A, 3B). Thus the presence of either analogue displaced urea from the gill tissue.

**Figure 4:**
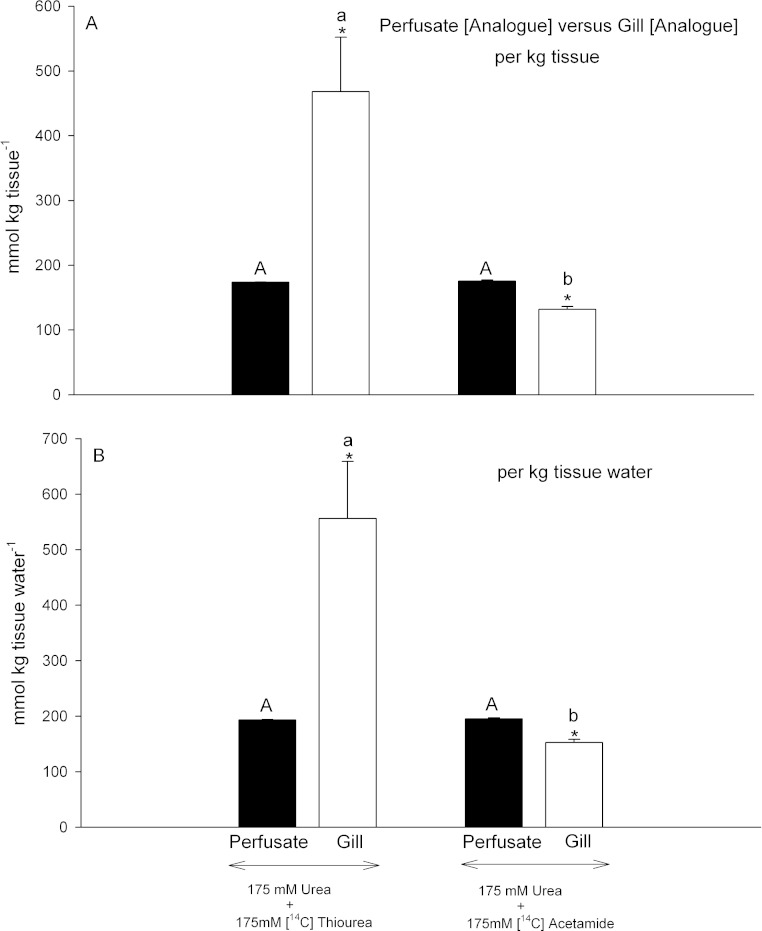
Analogue concentrations in the perfusate versus those measured simultaneously in gill tissue. Black bars represent concentrations in the perfusate; open bars represent concentrations in the gill tissue. In (A), concentrations are expressed on a per kg tissue or perfusate basis; in (B) concentrations are expressed on a per kg tissue H_2_O or perfusate H_2_O basis. The treatment (perfusion saline) is noted below the bars: 175 mmol L^−1^ urea + 175 mmol L^−1^ [^14^C]thiourea (*N* = 6); 175 mmol L^−1^ urea + 175 mmol L^−1^ [^14^C]acetamide (*N* = 6). Means ± 1 SEM. Perfusate means bearing the same capital letters are not significantly different (*P* < 0.05); gill tissue means bearing the same lower case letters are not significantly different (*P* < 0.05); asterisks indicate gill tissue mean significantly different (*P* < 0.05) from corresponding perfusate mean.

However the two analogues behaved very differently with respect to their own accumulation in gill tissue. Gill tissue concentrations of [^14^C]thiourea, were extremely high, about 2.8-fold those of the perfusate regardless of whether the data were expressed on a per kg tissue basis (Fig. 4A) or a per kg tissue H_2_O basis (Fig. 4B). Thus they were also significantly higher than the simultaneously measured gill [urea] (Figs. 3A, 3B) by comparable factors (2.9- fold). In contrast, gill tissue [^14^C]acetamide concentration was 25% lower than perfusate [^14^C]acetamide concentration (Fig. 4A), and this difference remained significant (22% lower) when the data were expressed on a per kg tissue H_2_O basis (Fig. 4B). Thus acetamide did not equilibrate between perfusate and tissue, and indeed tissue [^14^C]acetamide concentrations (Figs. 4A, 4B) also remained below tissue urea concentrations (Figs. 3A, 3B) by about 20%.

### The water content of gill tissue and its distribution

The water content of gills perfused with Control saline 1 was similar to that *in vivo*, and to that in gills perfused with Control saline 2 (Table 1). However the latter was significantly below the *in vivo* value by about 2.5%. Water contents were significantly higher by 3–6% in the two analogue treatments relative to all others, but there was no difference between gills perfused with 175 mmol L^−1^ thiourea plus 175 mmol L^−1^ urea *versus* 175 mmol L^−1^ acetamide plus 175 mmol L^−1^ urea.

Extracellular (ECFV) and intracellular (ICFV) volumes were measured only in preparations perfused with Control saline 2. Based on the distribution of the extracellular marker [^3^H]methoxy inulin, the tissue water was divided into an extracellular compartment of 64.79 ± 2.88 (5)% and an intracellular compartment of 35.24 ± 2.88% (5)%; absolute values are reported in Table 1.

## Discussion

### Overview

Overall, the data are consistent with the presence of an active urea back-transporter in the gill cells which is competitively inhibited by the urea analogues thiourea and acetamide. Our original hypotheses that the analogues would increase urea efflux rates, would displace urea from the gill cells, and would be excreted at comparable rates to urea when presented at equimolar concentrations in the perfusate were all confirmed. Our hypothesis that gill cell urea concentration would be buffered from changes in extracellular urea concentration was also confirmed, and helped explain the lack of correspondence between plasma urea concentration and urea efflux rate seen in this and previous investigations (see Introduction). However, our hypothesis that gill cell urea concentrations would be much lower than the extracellular concentrations was disproven; indeed gill cell urea concentrations appear to be normally very close to plasma concentrations both *in vivo* and in the perfused head preparation. As such, these results provide new evidence the apical membrane as the limiting factor in maintaining gill urea impermeability, and raise the prospect that a back-transporter may also operate at the apical membrane.

### Urea efflux rates

Mean urea efflux rates (∼ 450 µmol kg^−1^ h^−1^ ) from the head preparation when perfused with Control saline 1 were at the high end of the range (182–480 µmol kg^−1^ h^−1^ ) reported by Pärt, Wright & Wood (1998) for various control series in a very similar preparation. They were also slightly higher than rates (250–400 µmol kg^−1^ h^−1^ ) typically measured *in vivo* (Wood, Pärt & Wright, 1995; Wood et al., 2005; Kajimura et al., 2006) on *Squalus acanthias* from the same source. Our results showing only a small, non-significant drop in urea efflux rate (by about 35%) when perfusate urea was reduced by 50% (Control saline 2) were also in agreement with very similar results of Pärt, Wright & Wood (1998), and are explained, and least in part, by the very small measured change (22% drop) in gill tissue urea concentration. Thus gill urea concentration, and consequently urea efflux rates, appear to be buffered from changes in extracellular urea concentration.

In contrast, a major difference from the results of Pärt, Wright & Wood (1998) was our finding that equimolar thiourea markedly increased urea efflux rates. The likely explanation is that Pärt, Wright & Wood (1998) changed over the perfusate from 175 mmol L^−1^ urea to 175 mmol L^−1^ urea plus 175 mmol L^−1^ thiourea for a period of only 10 min, whereas we perfused continuously with the latter saline for 45 min. Indeed we found that urea efflux rates were usually unstable during the initial 10 min of perfusion with any solution. It is also noteworthy that 45 min perfusion with equimolar acetamide, another competitive analogue, had a very similar effect. These data are consistent with the *in vivo* findings of Wood, Pärt & Wright (1995) that thiourea and urea infusions increased branchial urea efflux rates in intact dogfish. However, it is more difficult to rationalize these results with the lack of significant effect of 148-fold excesses of thiourea and acetamide on urea transport rate in gill basolateral membrane vesicles (Fines, Ballantyne & Wright, 2001). As argued below, the most likely explanation is that thiourea and acetamide were acting on a different back-transport system in the current experiments.

### Gill urea and analogue concentrations

Contrary to earlier supposition that gill cell urea concentrations would be much below extracellular concentrations (Pärt, Wright & Wood, 1998; Fines, Ballantyne & Wright, 2001; Hill et al., 2004), the present measurements suggest that they are almost in equilibrium when expressed on a per kg water basis. Of course this conclusion depends to some extent on water distribution between ECFV and ICFV, which was measured only in Control series 2. In Table 2, we have applied the same ECFV and ICFV values to all the experimental series, so as to estimate mean intracellular urea and analogue concentrations in the gill cells. The calculation is based on the measured extracellular and gill tissue urea concentrations and water contents, and the assumption that the same ECFV applies to all treatments. Thus measured differences in water content, associated mainly with the analogue treatments (Table 1), would be due entirely to changes in ICFV. Nevertheless, model calculations, using the opposite assumption, that differences in water content were due entirely to changes in ECFV, yielded very similar values, indicating that the estimates are robust.

**Table 2:** Intracellular and extracellular concentrations of urea and analogues in gill tissue.

Treatment	Intracellular	Extracellular
	(mmol kg H_2_O^−1^ )	(mmol kg H_2_O^−1^ )
**Urea Concentrations:**		
*In vivo*	337	443
Control Saline 1 (350 mmol L^−1^ Urea)	409	385
Control Saline 2 (175 mmol L^−1^ Urea + 175 mmol L^−1^ Mannitol)	419	214
[^14^C]Thiourea (175 mmol L^−1^) + Urea (175 mmol L^−1^)	185	189
[^14^C]Acetamide (175 mmol L^−1^) + Urea (175 mmol L^−1^)	171	194
**Analogue Concentrations:**		
[^14^C]Thiourea (175 mmol L^−1^) + Urea (175 mmol L^−1^)	1135	194
[^14^C]Acetamide (175 mmol L^−1^) + Urea (175 mmol L^−1^)	87	195

**Notes.**

See text for calculation details.

These calculations (Table 2) suggest that *in vivo*, intracellular urea concentration is about 76% of extracellular urea concentration, and this rises to approximate equality in preparations perfused with Control saline 1. When extracellular urea is approximately halved, in perfusions with Control saline 2, intracellular urea remains unchanged. However, when equimolar thiourea or acetamide are present in the perfusion medium, then intracellular urea concentrations are reduced by about 55%. Thus in the absence of the analogues, gill intracellular urea is well-regulated in the face of a large decrease in extracellular urea, but the presence of either analogue markedly depresses intracellular urea concentration.

These calculations also indicate that intracellular thiourea concentrations are remarkably 6-fold higher than extracellular concentrations at the end of 45 min perfusion. This is not necessarily due to an active retention mechanism. Similar biomagnification has been seen in other systems such as the rat lung, and is probably explained by binding of the sulfhydryl moiety on thiourea to cellular membrane proteins (Hollinger, Giri & Hwang, 1976; Hollinger & Giri, 1990). Adsorption is unlikely to occur for either urea or acetamide, which do not contain sulfhydryl moieties. In contrast to thiourea, intracellular acetamide concentrations remained well below extracellular acetamide levels, as well as below intracellular urea levels, despite the ability of acetamide to displace intracellular urea. Based on both urea efflux rates and acetamide efflux rates, this analogue is a more effective blocker of the urea retention mechanism than thiourea, yet appears to be poorly reabsorbed itself. It is clear that urea transporters in different systems may vary both in their affinity for analogues, and in their transport capacity for analogues. See for example the studies on two different urea transporters in gills and kidney of an unusual ureotelic teleost, the gulf toadfish (*Opsanus beta*) by McDonald et al. (2000) and additional examples cited therein. In the kidney of *Squalus acanthias*, an apparent Na^+^-linked active urea reabsorption mechanism transports acetamide, but to a lesser extent than urea, yet it does not transport thiourea (Schmidt-Nielsen & Rabinowitz, 1964; Schmidt-Nielsen, Truniger & Rabinowitz, 1972).

### Implications and a new proposal

The present results clearly support the concept of an active urea back-transport mechanism in the gills, reducing effective urea permeability. However, while not discounting the presence of such a back-transporter and an unusual lipid composition in the basolateral membranes (Fines, Ballantyne & Wright, 2001), they return the focus to the apical membranes as the limiting barrier (Pärt, Wright & Wood, 1998; Hill et al., 2004). It is difficult to see how the basolateral membranes could be limiting when intracellular urea concentrations are close to extracellular levels. We suggest this dichotomy could be explained by the presence of a back-transporter on the apical membranes, actively scavenging urea from the boundary layer as it leaks out. The primary effects of thiourea and acetamide in the present study would have been due to competition for this apical back-transporter, thereby increasing urea efflux and reducing intracellular urea concentration. There is undoubtedly a strong inwardly directed Na^+^ gradient from the external seawater across the apical membranes that could power such a mechanism by co-transport, as in the dogfish kidney (Schmidt-Nielsen, Truniger & Rabinowitz, 1972), and unidirectional Na^+^ influx rates are many-fold higher than urea efflux rates in other elasmobranchs (Payan & Maetz, 1973; Carrier & Evans, 1972; Haywood, 1974; Bentley, Maetz & Payan, 1976). To our knowledge, this possibility has never been investigated.
